# Lymphedema attributed to sirolimus

**Published:** 2013-07-01

**Authors:** Mohammad-Reza Ardalan

**Affiliations:** ^1^Chronic Kidney Disease Research Center, Tabriz University of Medical Sciences, Tabriz, Iran

**Keywords:** Immunosuppression, Sirolimus, Transplantation

**Implication for health policy/practice/research/medical education:**

An inhibitor of the mammalian target of sirolimus (mTOR) is used as an agent for immunosuppression of solid organ transplantation. Its advantages include lack of nephrotoxicity and a lower incidence of post-transplant cancers and thrombotic microangiopathy. Delayed wound healing, proteinuria and pulmonary infiltration are reported complications of sirolimus.

An inhibitor of the mammalian target of sirolimus (mTOR) is used as an agent for immunosuppression of solid organ transplantation. Its advantages include lack of nephrotoxicity and a lower incidence of post-transplant cancers and thrombotic microangiopathy. Delayed wound healing, proteinuria and pulmonary infiltration are reported complications of sirolimus (1-3). A 26-year-old man developed extensive severe right lower limb edema five years after renal transplantation for primary focal segmental glomerulosclerosis–associated end-stage renal disease. Six months before beginning of this symptom, the cyclosporine was replaced with sirolimus (sirolimus) for prevention of chronic allograft nephropathy development. He was also taking mycophenolate mofetil, prednisolone and metoprolol. Doppler study of lower extremities revealed normal venous drainage. His serum creatinine level was 1.1 mg/ dl, hemoglobin was 13 mg/dl and serum albumin level was 4.5 g/dl. There was no family history of lymphedema. Damage or removal of regional lymph nodes, lymphatic radiation, infection, or tumor invasion, we also exclude the filariasis, that is a direct lymphatic invasion by the parasite Wuchereria bancrofti. He also had no history of lipectomy, burns, and scar excision. Other causes of edema, such as congestive heart failure, proteinuria and hepatic insufficiency were excluded (4,5).

Chest radiography and echocardiography revealed no pleural or pericardial effusion. An abdominal ultrasound scan showed no ascitis or any local fluid collection or lymphadenopathy. With the clinical diagnosis of sirolimus induced lymphedema, sirolimus was stopped and cyclosporine A restarted. The diagnosis of sirolimus induced lymphedema is usually made with a thorough history and physical examination. However one year after discontinuation his lymphedema was still persisted and does not improved (Figure 1).

**Figure 1:**
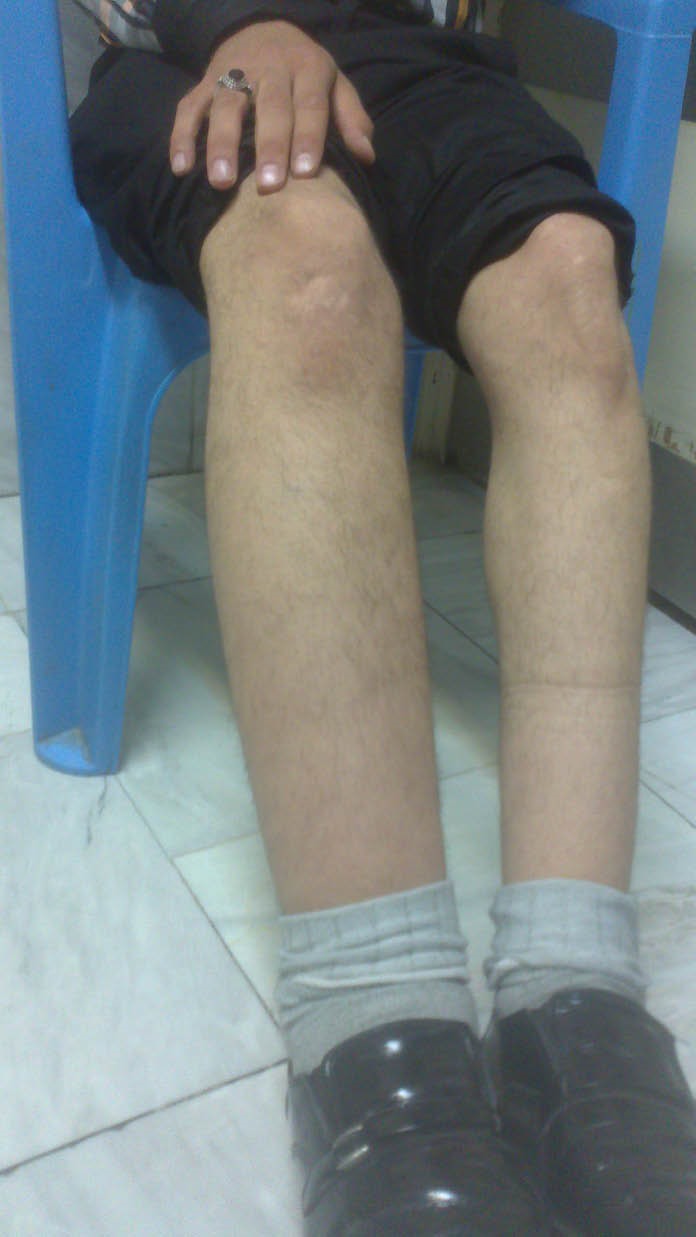
A 26-year-old man with severe right lower limb edema six months after starting sirolimus

Lymphedema is a debilitating progressive condition. It is a rare complication of sirolimus and only a few cases has been reported. Sirolimus potently inhibits vascular endothelial growth factor (VEGF)-C driven proliferation and migration of lymphatic endothelial cells (4-6). Full awareness about the rare complication of sirolimus help us to use it properly and safely. When there is a temporal relation between initiation of the medicine and symptom onset we should consider the possibility of sirolimus induced lymphedema. However in each instances the possibility of underlying malignancy, previous surgery and radiation of lymphatic system should be considered and ruled out. As in our reported case, because of devastation of lymphatic vessel lymphedema due to sirolimus, could persist for years after the drug discontinuation.

## Author’s contribution

MRA is the single author of the paper.

## Conflict of interests

The author declared no competing interests.

## Ethical considerations

Ethical issues (including plagiarism, misconduct, data fabrication, falsification, double publication or submission, redundancy) have been completely observed by the author.

## Funding/Support

None.
